# How oxygen deficiency in the Baltic Sea proper has spread and worsened: The role of ammonium and hydrogen sulphide

**DOI:** 10.1007/s13280-022-01738-8

**Published:** 2022-06-23

**Authors:** Carl Rolff, Jakob Walve, Ulf Larsson, Ragnar Elmgren

**Affiliations:** 1 Stockholm University Baltic Sea Centre, Stockholm University, 106 91 Stockholm, Sweden; 2 Department of Ecology, Environment and Plant Sciences, Stockholm University, 106 91 Stockholm, Sweden

**Keywords:** Anoxia, Baltic, Deoxygenation, Hypoxia, Inflow, Oxygen status

## Abstract

Even large inflows of oxygen-rich seawater to the Baltic Proper have in recent decades given only short-lived relief from oxygen deficiency below the halocline. We analyse long-term changes in oxygen deficiency, and calculate the “total oxygen debt” $\Sigma$OD, the oxygen required to oxidize the hydrogen sulphide (H_2_S) and ammonium (NH_4_) that builds up during stagnation periods. Since the early 1990s, oxygen below 65m has gradually decreased during successive stagnation periods, and the $\Sigma$OD has increased, with NH_4_ more important than previously recognised. After the major inflow in 2014, the Baltic Proper $\Sigma$OD has reached its highest level so far. The gradual shift of the $\Sigma$OD to shallower sub-halocline waters in the western and northern basins has increased the risk of periodic coastal hypoxia and export of hypoxic water to the Bothnian Sea. The potential for inflows large enough to more than eliminate the $\Sigma$OD seems limited in the near term.

**Supplementary Information:**

The online version contains supplementary material available at 10.1007/s13280-022-01738-8.

## Introduction

In the past half century, oxygen concentrations have declined in many coastal waters globally (Diaz and Rosenberg 2008; Breitburg et al. 2018). Nutrient enrichment, which stimulates organic matter loading, has been a major driver, but effects of climate change are of increasing concern, and influence water renewal, oxygen saturation levels and respiration rates (Cloern 2001; Fleming-Lehtinen et al. 2008; Conley et al. 2009; Carstensen et al. 2014; Andersen et al. 2017; Meier et al. 2018). One of the largest coastal ‘dead zones’, where oxygen levels are now too low for most multicellular animal life is in the Baltic Sea (Conley et al. 2009, Fig. S1).

The Baltic Sea is a semi-enclosed, shallow (mean depth 55 m), brackish waterbody with negligible tides. Its southern part, the Baltic Proper, has a permanent halocline at approximately 60 to 80 m depth and irregular deep water renewal by inflows from the North Sea. Its deep water is therefore naturally prone to periodic hypoxia or anoxia (Pettersson 1894; Fonselius 1963, 1981; Fonselius and Valderrama 2003; Conley et al. 2009; Zillén and Conley 2010; Savchuk 2013; Carstensen et al. 2014; Andersen et al. 2017; Papadomanolaki et al. 2018; Almroth-Rosell et al. 2021; Kouts et al. 2021). The present situation, with the area affected by hypoxia exceeding 80 000 km^2^ (Hansson et al. 2019; Almroth-Rosell et al. 2021), almost twice the area of Denmark (Fig. S2, Fig. S3), seems, however, to be unprecedented since at least 1500 CE (Hansson and Gustafsson 2011).

In the last hundred years, the nutrient load to the Baltic Sea has increased substantially, primarily from agriculture and sewage (Larsson et al. 1985; Gustafsson et al. 2012 and references therein). Eutrophication is generally considered the main reason for the present severe oxygen deficiency of the Baltic Proper (Larsson et al. 1985; Conley et al. 2002; Carstensen et al. 2014 and references therein). Model estimates suggest, however, that the nutrient loads peaked in the 1980s, and that the total phosphorus load has since then been roughly halved and the nitrogen load reduced by more than a third (Gustafsson et al. 2012; HELCOM 2018a). The oxygenation of the deep water in the Baltic Proper is also strongly affected by intermittent inflows of oxygenated seawater from the North Atlantic that influence its salinity, temperature and stratification (Meier et al. 2006; Matthäus et al. 2008; Reissmann et al. 2009; Mohrholz 2018). The relative importance of eutrophication and inflow dynamics for deep water oxygenation still remains unclear (e.g. Gerlach 1994; Zillén et al. 2008; Kabel et al. 2012; Carstensen et al. 2014).

Several quantitative indicators for describing the severity of oxygen deficiency in the Baltic Sea are in use (e.g. MacKenzie et al. 2000; Conley et al. 2002; Gustafsson and Stigebrandt 2007; Carstensen et al. 2014; Andersen et al. 2017; HELCOM 2018b; Almroth-Rosell et al. 2021). They scale differently to driving factors, which potentially affects the setting of targets for abatement measures. These indicators include (1) the oxygen or hydrogen sulphide concentration in the near-bottom water of the central Gotland basin, (2) the areal extent of sediments overlain by hypoxic and anoxic waters, (3) the volume of hypoxic or anoxic water, (4) the volume of water considered to allow successful cod spawning and (5) the oxygen deficit relative to full oxygen saturation (HELCOM 2018b). The first of these indicators focuses overly on conditions in the deepest part of the central basin. The area and volume indicators are affected by scaling problems caused by the bottom topography. The area covered by hypoxic and anoxic waters does not take into account the degree of accumulation of hydrogen sulphide (H_2_S) and ammonium (NH_4_^+^, below denoted NH_4_) and looses sensitivity when oxygen conditions have deteriorated severely. As the depth to the oxycline decreases, increasing volumes of water are required to cover an additional unit of bottom area because of the topography, with the deepest areas being relatively small. Using volume of hypoxic or anoxic waters avoids the latter problem, but not the first. Since open waters above the halocline are generally well ventilated, the halocline limits how large areas or volumes that can become hypoxic or anoxic. The HELCOM indicator overcomes these problems using the oxygen deficit relative to oxygen saturation, but neglects the NH_4_ contribution to the oxygen debt. The NH_4_ contribution has been noted but not studied in detail (Carstensen et al. 2014; Stigebrandt and Andersson 2020). By using the mean oxygen deficit below the halocline, the HELCOM indicator implicitly assumes that changes are equally important regardless of depth. However, oxygen in the upper layers of the deep water may not necessarily remove a debt in deeper layers. Moreover, the geographical distribution of the oxygen debt has largely been overlooked. Inflows initially improve conditions in the southern and eastern basins, whilst pushing up poorly oxygenated water to shallower depths in northern and western basins. Such redistribution of the oxygen debt may have important consequences for its further development and for coastal upwelling of poorly oxygenated deep water.

In this study, we evaluate the development of Baltic deoxygenation since 1970 by calculating the time series of the total amounts of oxygen, H_2_S and NH_4_ below 65 m. We then calculate how much oxygen is needed to eliminate all H_2_S and NH_4_ below this depth, define this as an oxygen debt, and discuss the utility of this approach for describing the oxygenation state of the Baltic. We also investigate how the distribution of this oxygen debt has changed over time amongst basins and depth layers. Our study is entirely data-driven and based on publicly available national monitoring data from Sweden and Finland.

## Water exchange with the Ocean

The riverine freshwater inflow to the Baltic drives an estuarine circulation that is active the whole year, but most intense during peak discharge in spring and summer. Brackish surface water leaves the Baltic Sea for the Kattegat through the Danish Straits. The normal inflow through the Danish Straits is a mixture of Baltic Proper surface water and fully marine North Sea surface water and flows into the Baltic at depths below the halocline, but is mostly not dense enough to replace the deepest water. Replacement of the deep water of the central basins occurs only sporadically, usually during winter storms (Schinke and Matthäus 1998). This requires an inflow of cold seawater that is denser than the Baltic Proper deep water, even after the salinity of the inflow is reduced by mixing with the resident deep water by entrainment. How complete the replacement becomes and how far into the Baltic it reaches depends on the volume, salinity and temperature of the inflow. Large inflows that ventilate the deep water are termed Major Baltic Inflows (MBIs) and appear to have become less frequent and more irregular since the early 1980s (Fig. S4), affecting the oxygen status of the Baltic Proper deep water (Fischer and Matthäus 1996).

**Fig. 1:**
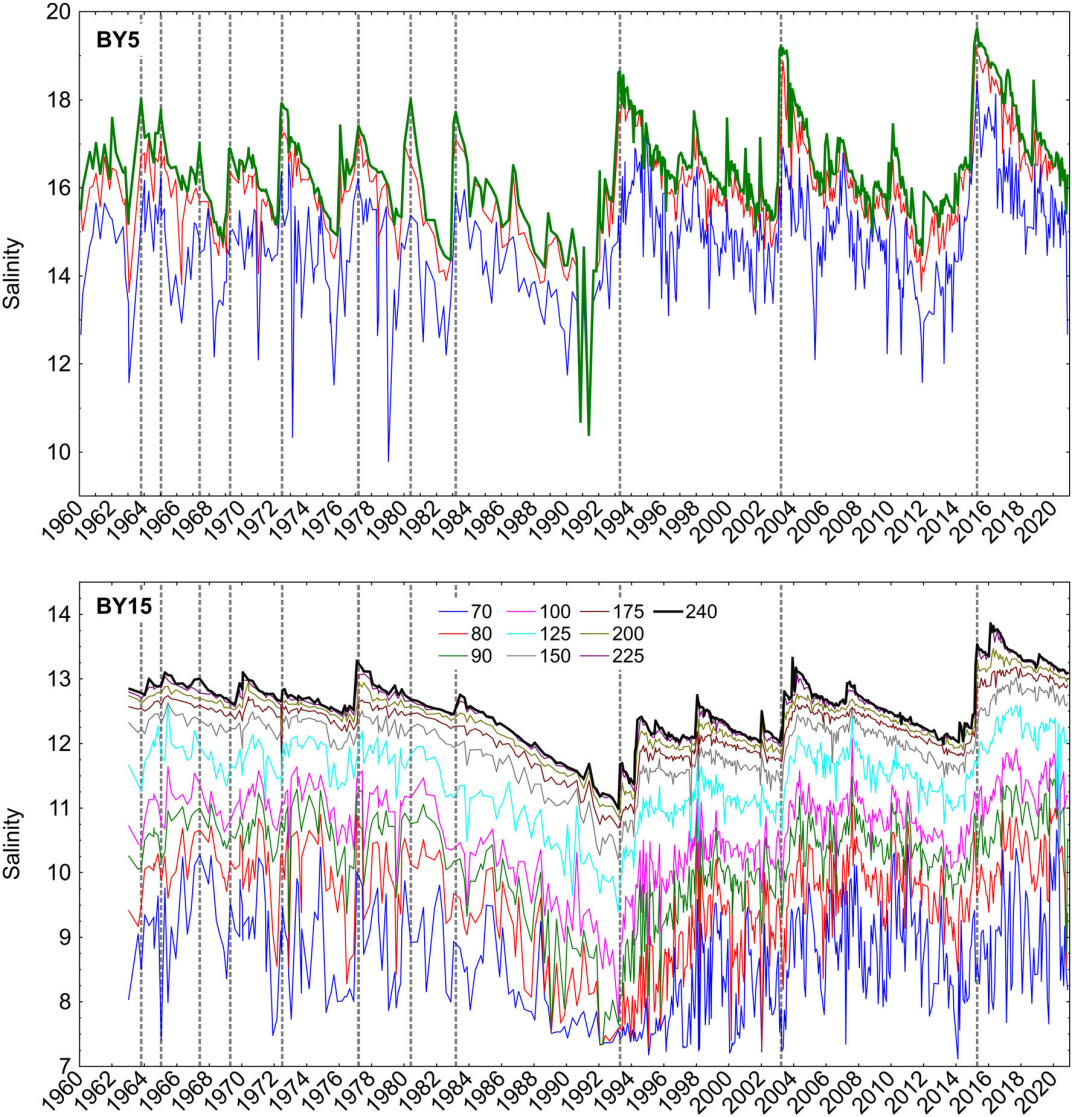
Deep water salinities in the Bornholm Deep station BY5 (upper panel) and the Gotland Deep station BY15 (lower panel). Inflows with a clear impact on deep water salinity at BY5 are indicated by dotted lines, also for BY15. Swedish monitoring data from the SMHI database SHARK

The change in inflow regime reported by Fischer and Matthäus (1996) was questioned by Mohrholz (2018), who argued that much of the perceived change in inflow regime was caused by lack of data between 1976 and 1991, in combination with methodological problems. Mohrholz instead found a decadal variability of MBIs with a time scale of 25–30 years and identified periods with reduced MBI frequency. A description of these inflows is beyond the scope of this study but the salinities of the deep waters in the southern Bornholm Deep (NE of Bornholm) and the central Gotland Deep (E of Gotland) strongly support that MBIs have been less frequent after 1984 (Fig. 1). The Bornholm Deep (station BY5) is the first basin reached by inflowing high-density seawater. Here, the periods 1960–1984 and 1985–2018 show markedly different patterns of deep water salinity. In the earlier period, major salinity peaks appeared at intervals of ~ 3 to 5 years, whereas the time between large peaks in the latter period is almost 12 years, with smaller peaks in between. In the later period, the large inflows caused a greater salinity increase. This was likely caused by the long period of salinity decline preceding the inflow, rather than by different inflow volumes, if the relative sizes of these are correctly estimated (Fig. S4). These differences are not as clearly seen in the central Gotland Deep, but the salinity increase caused by inflows was still generally larger in the later period.

The MBIs that ventilate the deep water carry large amounts of salt, thereby increasing the density difference across the halocline. This strengthening of the halocline hampers vertical mixing, reducing ventilation of the deep water (Gerlach 1994). The inflows are therefore potentially a two-edged sword that may improve oxygen conditions in the short term, but worsen them in the long term, should their frequency decrease. The frequency of the inflows is thus crucial for deep water oxygenation. If MBIs are frequent enough they will maintain good oxygen conditions but if they are large and followed by long periods without inflows, they may cause deteriorating conditions by counteracting vertical mixing and upwelling by storm events.

**Fig. 2:**
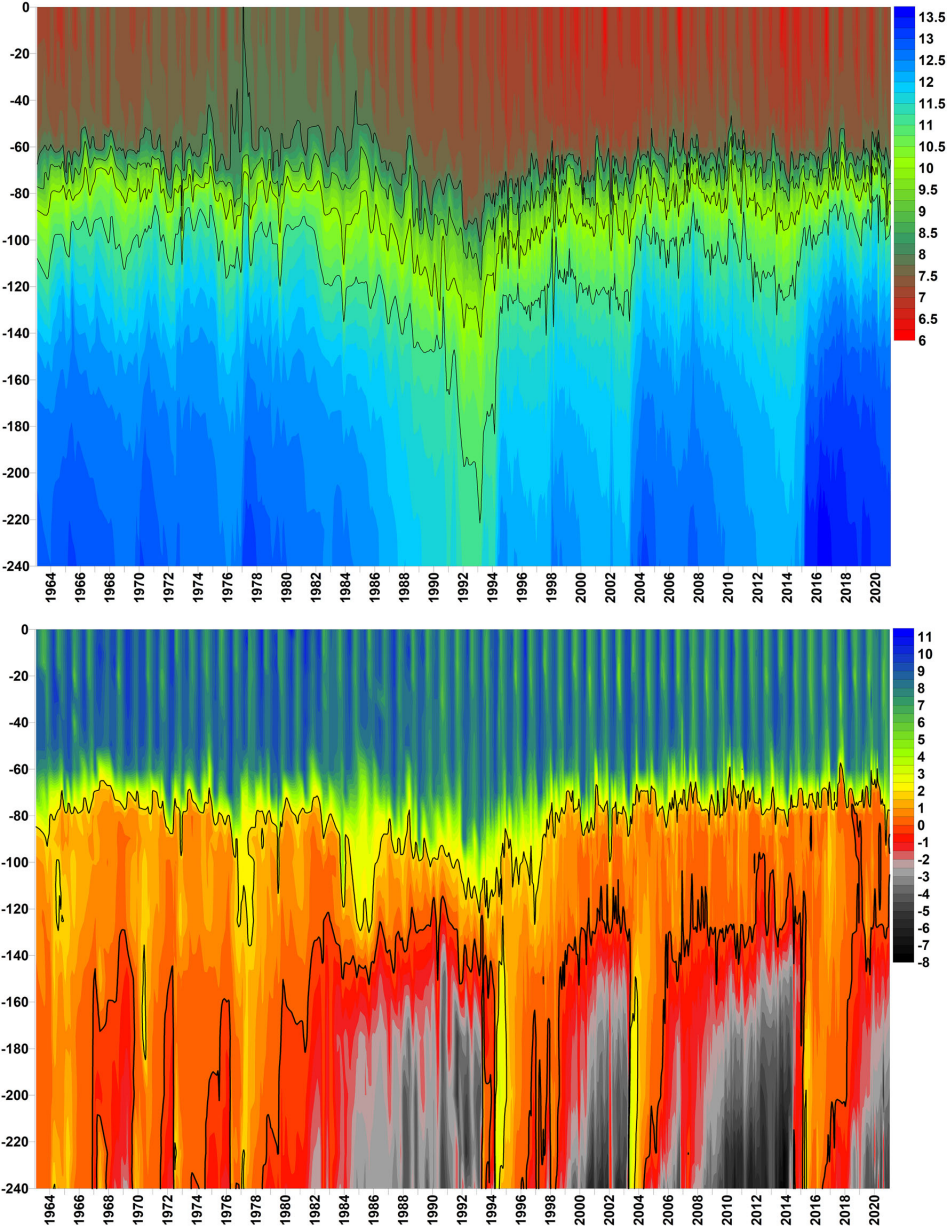
Isopleths versus depth (metres) at station BY15 for salinity (Top, lines indicate 8, 9, 10 and 11) and oxygen in mL L^−1^ (Bottom, thin line indicates oxygen concentration < 2 mL L^−1^ and thick line < 0 mL L^−1^). Swedish monitoring data from the SMHI database SHARK

MBIs can change the depth distribution of salinity and oxygen drastically, as is well illustrated by the central basin BY15 (Fig. 2). The major inflow of 1993 ended a long stagnation period, with decreasing salinity, weakening of the halocline and accumulation of H_2_S in the deep water. After the inflow the entire water column was oxygenated down to the bottom at 240 m depth, and the Baltic Proper bottom area covered by hypoxic or anoxic water reached a minimum (Fig. 2, Fig. S3). The preceding period (c. 1982–1993) with a deep, weakening halocline was also a period of decreasing oxygen in the deepest water, contradicting the assumption that a weak halocline in itself is a sufficient prerequisite for oxygenated conditions below the halocline (Fig. 2). However, this period also saw the maximal annual nutrient load on the Baltic (Gustafsson et al. 2012), which may have caused sedimentation of organic matter to be at its highest during this period. The deep, weak halocline before 1993 allowed intermediate inflows and mixing to ventilate a large volume of water at intermediate depths. This resulted in a maximum of oxygen in the waters below 65 m in early 1993, in spite of the simultaneously high levels of H_2_S in the deepest water, later eliminated by the 1993 inflow. After 1993, there were two major, distinct decadal periods of decreasing salinity and increasing H_2_S concentrations, ended by MBIs in 2003 and 2014, with minor inflows in between (Fig. 1). Another such cycle seems to follow the MBI in 2014, with additional minor inflows thereafter.

## Materials and Methods

### Data sources

Data were downloaded from the publicly available national Swedish database SHARK (stations BY1 to BY38, Table S1) of the Swedish Meteorological and Hydrological Institute,^1^ and Finnish national data for ICES stations LL12, LL7 and LL3A, from the ICES database.^2^ The data were collected in national monitoring programs carried out by the same national organisations for the entire study period.

### Basin division

**Fig. 3:**
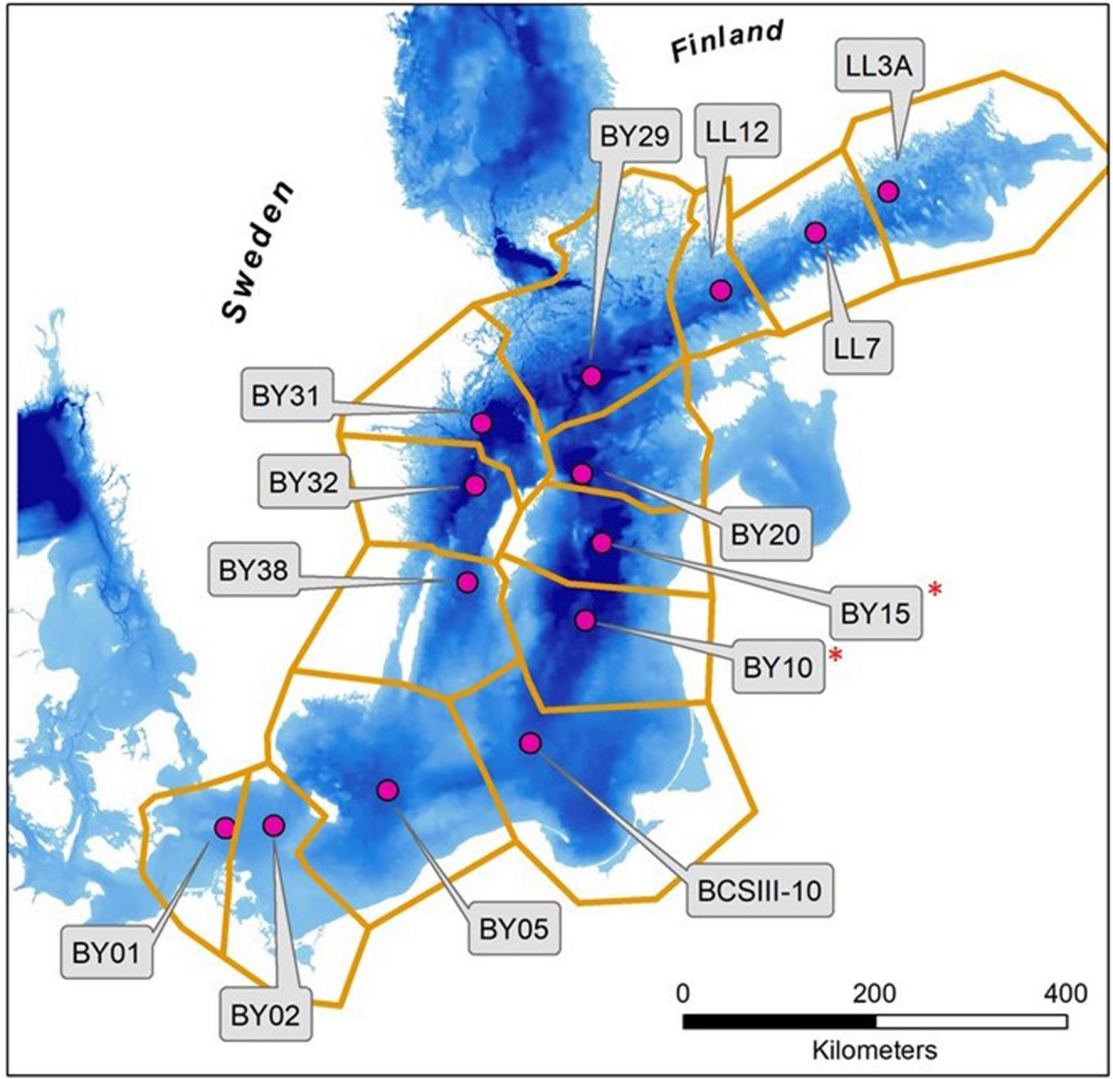
Map of stations in the programs and their associated basins. * In the text, basins BY10 and BY15 are treated as one and called BY15

Oxygen (O_2_), hydrogen sulphide (H_2_S) and ammonium (NH_4_) amounts were calculated for basins adapted to the national monitoring programs of Sweden and Finland, using the station names in the SHARK and ICES databases (see also below). Basins are generally named with two digits e.g. station BY5 corresponds to basin BY05, etc. (Table S1). The border between basins was generally defined so that each monitoring station represents the water closest to that station (Voronoi partition, Fig. 3, Table S1). In several cases, a strict application of this rule was not in agreement with the topography and minor adjustments to the basin borders were made to keep topographically continuous deep water volumes connected. Some basin borders are therefore closer to one station than to its neighbours (e.g. for basins BY15, BY20, BY32 and BY38 in Fig. 3). This ensures that a deep water volume in a basin is represented by the station likely to be most representative of that deep water volume but also causes some water volumes at more shallow depths to be represented by a station further away than the closest station. Since sampling at BY10 started later than at the other stations, BY15 + BY10 is treated as one basin (using only data from BY15), henceforth called BY15. Because of its central position, large volume and relatively slow responses, this basin east of Gotland is often used to illustrate large-scale and long-term biogeochemical processes in the Baltic Proper (eg. Conley et al. 2002; Vahtera et al. 2007; Snoeijs-Leijonmalm et al. 2016; Savchuk 2018).

The hypsographs are based on the publicly available HELCOM topographic grid “Bathymetry of the Baltic Sea (BALANCE)”, downloaded 2017-06-28.^3^ We also tested the alternative grid “Baltic Sea Hydrographic Commission, 2013, Baltic Sea Bathymetry Database version 0.9.3, downloaded 2016-10-14,^4^ but the differences were negligible.

The summed volume of water below 65 m in all basins is ~ 3 485 km^3^, with 34% of this in the central basin BY15 (BY15 + BY10), and only slightly less than 3% in basins LL12, LL7 and LL3 in the Gulf of Finland (Table 1). The basins BY15 and BY29 together have more than half of the total volume and therefore have a large effect on total estimated amounts of oxygen, H_2_S and NH_4_. Basins BY01 and BY02 have no depths below 50 m.

### Data treatment

On the vast majority of sampling occasions, physical and chemical variables (temperature, salinity, oxygen, H_2_S and NH_4_) were measured in profiles from surface to bottom at 0, 5, 10, 15, 20, 30, 40, 50, 60, 70, 80, 90, 100, 125, 150, 175, 200, 225, 250, 300, 400 and 440 m and in near-bottom water (Table S1). The total dataset contains 330 000 measurements and six thousand surface to bottom profiles. Where feasible, missing data were estimated by linear interpolation (se also Supplementary Information).

Hydrogen sulphide was converted to negative oxygen by the conversion: 1 H_2_S = 2 O_2_ or 1 µmol L^−1^ H_2_S = − 0.04478 mL L^−1^ O_2_ (Fonselius 1981). Negative oxygen was calculated only if H_2_S > 4 µmol L^−1^, since measurements below this level were considered uncertain. If a low oxygen concentration was measured and H_2_S < 4 µmol L^−1^ the oxygen concentration was used, but oxygen was assumed to be zero if no oxygen concentration was given and H_2_S < 4 µmol L^−1^. When assessing oxygen demand for NH_4_ oxidation, one tonne of NH_4_ nitrogen was assumed to require ~ 4.57 tonnes of oxygen for oxidation to nitrate (Table S2). Nitrite produced by the first step of nitrification could however also be partially processed by the anammox path, requiring only ~ 1.71 tonnes of oxygen per tonne of NH_4_ nitrogen.

### Methods of calculating oxygen debt

We have chosen to calculate total oxygen debt ($\Sigma$OD) as the oxygen required to fully oxidize both H_2_S and NH_4_ present in the deep water below 65 m (see below). We then calculate net oxygen by subtracting this oxygen debt from the oxygen present below 65 m. This net oxygen would result if the water below 65 m mixed fully, allowing H_2_S and NH_4_ to oxidise. This rarely happens because topography and density differences restrict mixing, allowing high deep water concentrations of H_2_S and NH_4_ to build up over time, even with excess oxygen present below the halocline (Fig. 2). Oxidation of the large amounts of H_2_S and NH_4_ that build up during stagnation therefore require the oxygen transport and mixing caused by MBIs. How much oxygen is consumed by oxidizing H_2_S and NH_4_ is currently not known, since the gross production of the latter is unknown.

Oxygen debt was calculated as the amount (metric tonnes) of H_2_S and NH_4_, expressed as negative oxygen, for each depth stratum of every basin. A depth stratum is defined upwards and downwards from a sampling depth to the midpoint between this and the next sampling depth. For e.g. 0, 5, 10, 15, 20 and 30 m the corresponding strata are [0, 2.5), [2.5, 7.5), [7.5, 12.5), [12.5, 17.5), [17.5, 25), [25, 35) metres, etc. The amounts were calculated simply as the product of concentration and volume of the corresponding stratum, assuming that the concentration found at the sampling depth was the same in the entire stratum. This introduces a slight overestimate since volume decreases with depth within each depth stratum. However, calculations made per metre, with linearly interpolated concentrations, differed negligibly.

To facilitate summing of substance amounts amongst basins with different seasonal resolution, daily linearly interpolated values were calculated for each substance in each stratum of each basin. This procedure compensates for most situations where occasional sampling dates are missing or dates differ substantially between stations. It also reduces unintended biases caused by variable sampling frequency when calculating averages or medians. Data before 1994 generally had a resolution of four to six sampling occasions per year, with all seasons represented, whilst data from 1994 on had at least monthly resolution.

### Halocline depth

**Fig. 4:**
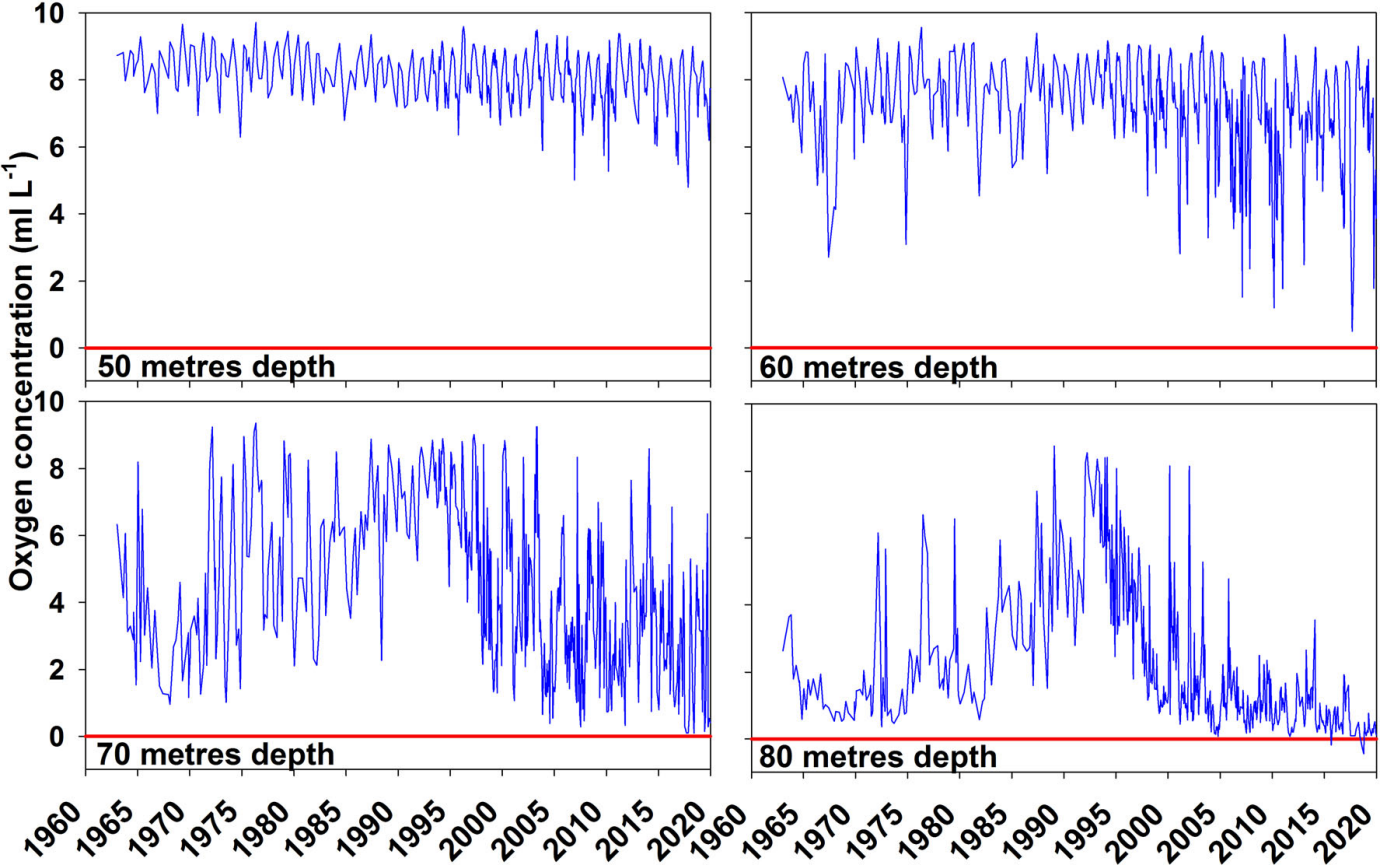
Oxygen concentration at 50, 60, 70 and 80 m depth at station BY15

**Fig. 5:**
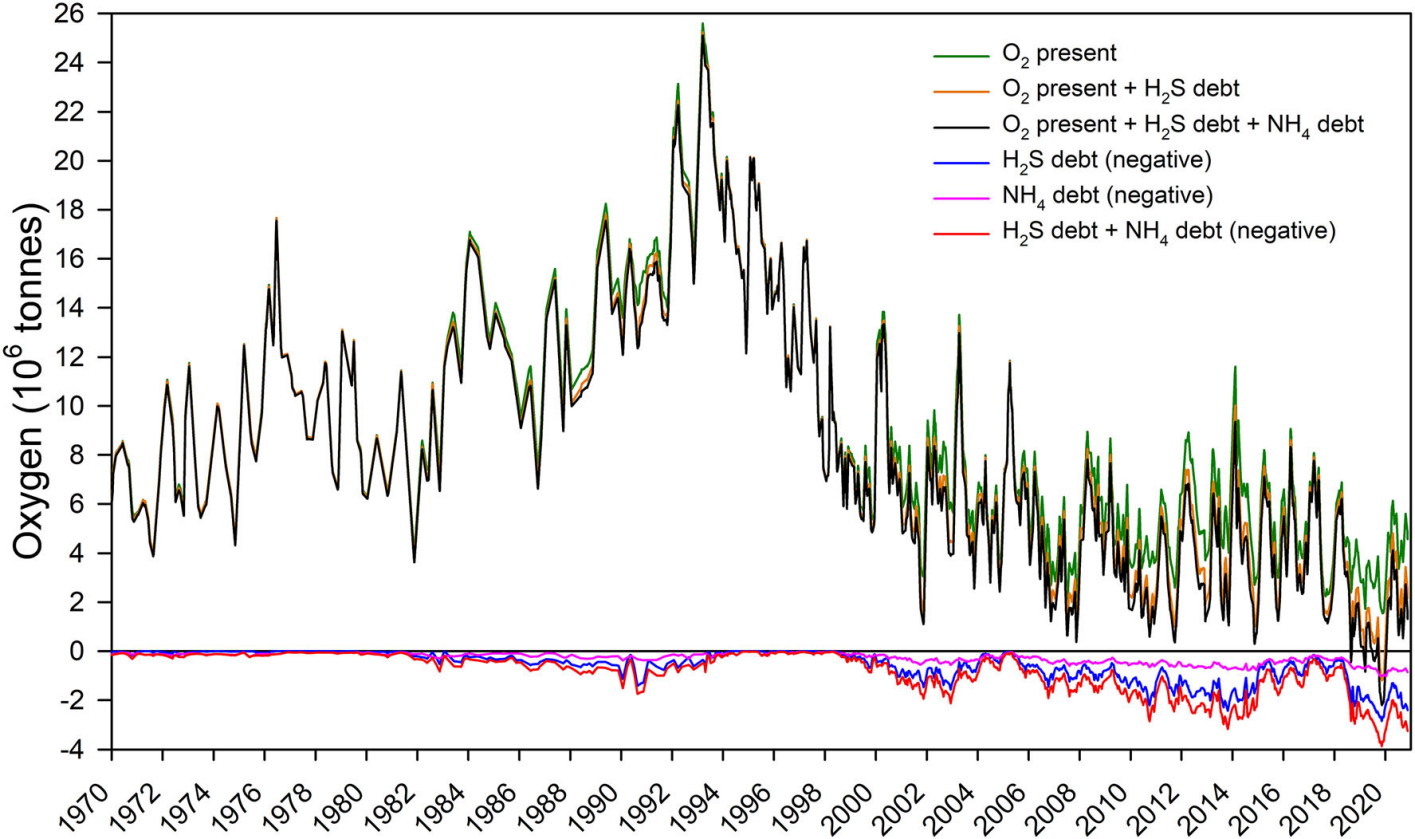
Different summed daily estimates of oxygen present (O_2_ present, O_2_ present + H_2_S debt, O_2_ present + H_2_S debt + NH_4_ debt) and oxygen debts (H_2_S debt, NH_4_ debt, H_2_S debt + NH_4_ debt), the latter shown as negative tonnes oxygen, in the B65 volume of basins BY05-BY38 (i.e. excluding BY01, BY02, LL12, LL7 and LL3). The high oxygen amounts in the 1990s were due to a deep and weak halocline (see “Methods”)

The depth of the halocline in the central part of the Baltic Proper can normally fluctuate between 60 and 80 m (e.g. Snoeijs-Leijonmalm et al. 2016; Almroth-Rosell et al. 2021) depending on basin, year, season, inflows and storm events (Fig. 2)***.*** For the purpose of this study, it was practical not to use the actual time-specific level of the halocline, since this would result in varying volumes for calculating oxygen present and oxygen debts. In addition, the halocline depth and thickness are difficult to estimate since the position of the density inflexion point and the depth interval of rapid density change can show considerable short-term variations. Water immediately above 70 m generally has oxygen present (Fig. 4) and very low concentrations of H_2_S or NH_4_, but large volumes per metre depth. Errors in exactly defining the halocline position would therefore introduce variation from volume as a variable and complicate interpretation of oxygen amounts without increasing precision in the estimate of $\Sigma$OD. A halocline at 70 m is a good approximation for all central basins and inclusion of shallower layers only marginally affect calculated $\Sigma$OD (see “Results”). Unlike the $\Sigma$OD, the estimate of net oxygen is affected by changes in halocline depth. The extreme values around 1990 were a result of an exceptionally deep and weak halocline, causing inclusion of oxygen-rich water below 65 m but above the halocline (Fig. 5). The concentration at 70 m then represents the uppermost layer of deep water (65–75 m) and the volume from 65 m to the bottom will below be referred to as “B65” and used as an approximation of “below the halocline”. The shallow basins BY01 and BY02 do not exceed 50 m depth and their halocline position is shallow and very variable. The volumes below the halocline in these basins are small and comparatively well oxygenated. They do not quantitatively affect estimates of summed oxygen debt and were excluded from the calculations. Sampling of the stations LL12, LL7 and LL3 was less seasonally complete than at the SMHI stations BY1-BY38, and 2020 data were not available when our calculations were made. They were therefore also excluded, after an estimate of the effect of including them up to August 2019 had been made (see “Results”).

## Results and discussion

### Available oxygen and oxygen debts

An intuitive measure of oxygen status is the actual amount of oxygen present in the water below 65 m (B65) (Fig. 5). In the 1970s the oxygen amount in B65 was around 6 to 10 million tonnes. Oxygen reached a maximum of around 26 million tonnes in the early 1990s caused by a deep and weak halocline and oxygenation of the entire water column in BY15 following the 1993 MBI. The amount of oxygen present in the B65 volume has since then gradually decreased and is now only 2.5 to 5 million tonnes as an annual average. An alternative measure of oxygen status is the oxygen amount present reduced by adding the oxygen debt caused by H_2_S, expressed as negative oxygen. This sum has gradually declined since the peak values in the early 1990s and fell below zero in 2019. When the oxygen debt caused by NH_4_ is added, the net oxygen amount in 2019 falls below minus 2 million tonnes. The estimated oxygen amounts (and net oxygen) are sensitive to drastic changes in halocline depth whereas $\Sigma$OD is not.

The oxygen status can also be illustrated by focussing only on the debts (here expressed as negative oxygen) caused by the presence of H_2_S and NH_4_, separately and summed as $\Sigma$OD (Fig. 5). During the stagnation periods following the major inflows of 2003 and 2014, $\Sigma$OD reached more negative values with a record of minus nearly 4 million tonnes in 2019. That large debts can build up, even though oxygen is simultaneously present in other depth strata below the halocline, shows that mixing is limited, so that oxygen present below the halocline cannot fully eliminate the debts in the deeper water. Such elimination requires external oxygen input. It is therefore clear that debts caused by H_2_S and NH_4_ can build up even though oxygen is present in the B65 volume.

The effect of limiting debt calculations to below 65 m (estimated from concentrations at 70 m and deeper) was tested by calculating the corresponding summed H_2_S and NH_4_ oxygen debts below 45 and 55 m. The differences were negligible (Fig. S5). Since this study focusses on the debts, they will graphically and in the text henceforward be given as positive values, except when compared to oxygen.

**Fig. 6:**
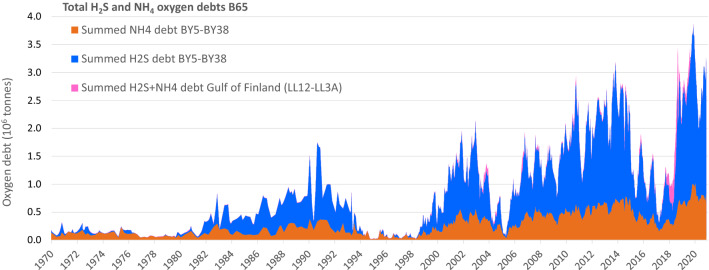
Debts as positive values (stacked areas) of H_2_S- (blue) and NH_4_ oxygen debts (brown) in the volume below 65 m (B65) of basins BY05, BCSIII-10, BY15, BY20, BY29, BY31, BY32 and BY38. The summed H_2_S and NH_4_ debts in B65 of stations LL12, LL7 and LL3 in the Gulf of Finland are added on top (purple). The extreme dip in H_2_S oxygen debt in 1990 is an artefact caused by missing H_2_S data for one date at BY15

The total Baltic Proper (BY05-BY38) oxygen debt ($\Sigma$OD) has increased over time (Fig. 6)*.* Before 1980, it was generally below 200 000 tonnes but has since progressively increased to over 3.5 million tonnes in 2019, with a yearly average of over 2.7 million tonnes since mid-2018.

Studies of oxygen debt have generally focussed on H_2_S, and neglected the debt caused by NH_4_. Stigebrandt and Andersson (2020) did, however, calculate an H_2_S plus NH_4_ oxygen debt for the Eastern Gotland Basin (approximately our basins BCSIII-10, BY15 and BY20). Their maximum debt estimate, just above 1.4 million tonnes of oxygen, is close to our value of about 1.5 million tonnes for these basins. The oxygen debt concept discussed by Carstensen et al. (2014) uses a variable halocline, relates to nutrient load and does not include NH_4_ and is therefore not directly comparable to ours.

### Oxygen debt caused by NH_4_ is potentially more persistent than that caused by H_2_S

Over time, the improvements in oxygen status caused by large inflows have become increasingly short-lived and the total oxygen debt ($\Sigma$OD) built up before the next inflow has increased. The longer periods between major inflows in 1985–2020 relative to 1960–1985 resulted in a larger build-up of H_2_S and NH_4_ oxygen debts, with less short-time variation for NH_4_ (Fig. 6). In periods of high $\Sigma{\text {OD}}$, NH_4_ typically contributes about 30% of $\Sigma$OD. When oxygenation is good, NH_4_ oxygen debt can be over 90% of $\Sigma{\text {OD}}$. The 1993 oxygenation event essentially eliminated both NH_4_ and H_2_S oxygen debts, but residual oxygen debts were observed following later inflows.

After the inflows of 2003 and 2014 approximately 30 and 40% respectively of the H_2_S oxygen debt and 55 and 50% of the NH_4_ oxygen debt remained, when calculated as ratios of annual means of debts in the year before the inflow and the second year after. When an inflow occurs, the elimination of NH_4_ by nitrification–denitrification or anammox seems to be slower and less complete than elimination of H_2_S, potentially reflecting the different thermodynamics of the NH_4_ and H_2_S reactions with oxygen. The NH_4_ oxygen debt thus appears to have a longer “memory” than the H_2_S debt. This, combined with the greater loss of oxygen during long stagnation periods, may partly explain why recent, relatively large inflows have provided only short-lived relief from hypoxia.

Whether NH_4_ is actually oxidized all the way to nitrate as the deep water is ventilated is difficult to evaluate quantitatively by budgeting, because of the highly dynamic nitrite+nitrate concentrations in the near-halocline waters. Ammonium can be oxidized to nitrate, be flushed out by replacement of water masses or be further transformed to N_2_ by nitrification–denitrification or the anammox pathway. If all NH_4_ oxidation would follow the anammox path, the calculated NH_4_ oxygen debts would be reduced by about 60% (Table S2). In the period 2000 to 2020, which includes the inflows of 2003 and 2014, summed nitrite+nitrate and NH_4_ (DIN) below 65 m (B65) in basins BY05-BY38 remained relatively constant around 250 000 tonnes nitrogen. This suggests that NH_4_ lost in connection with MBIs is oxidised mainly to nitrate using imported oxygen rather than being flushed out or processed by the anammox pathway. Comparing conditions during the year before an inflow with those two years later, the NH_4_ proportion of DIN shifted from 35 to 30% after the inflow of 2003 and from 56 to 30% after that of 2014. It is not known how much of the NH_4_ present after two years is new NH_4_, released from decomposing organic matter during those years, and how much remains from before the inflow.

Factors other than $\Sigma{\text {OD}}$ can potentially affect the oxygen status of the waters below the halocline. The temperature below the halocline is affected by the inflow regime, in the deep water generally decreasing during stagnation by heat loss and increasing by inflows of Kattegat surface water. The reduction in oxygen content at saturation in warmer seawater was estimated to about 0.5 mg·L^−1^ (0.35 ml L^−1^) over the past 115 years by Carstensen et al. (2014). Stigebrandt and Andersson (2020) estimated only a 3% oxygen reduction per degree of warming. Savchuk (2018) found an annual average temperature increase of 0.04 °C below 60 m in the Baltic Proper since 1979. The range of volume-weighted temperature fluctuation in the deep water (B65) of basin BY15 is ~ 3.5 degrees for the period 1970 to 2020. Increased rates of respiration at higher temperature can also contribute to the increasingly rapid deterioration of oxygen conditions after inflows. Warmer bottom waters after the stagnation period that ended in the 1990s is estimated to have increased deep water respiration by ∼20% (Carstensen et al. 2014). Other reduced substances, such as ions of iron and manganese, may constitute additional potential oxygen debts. However, a calculation based on published maximum concentrations of dissolved iron (Fe(II), 1.2 µmol L^−1^) and manganese (Mn(II), 15 µmol L^−1^) found in anoxic deep water in the BY15 basin (Neretin et al. 2003; Pohl and Fernández-Otero 2012) suggests that complete oxidation of these ions would require up to about 0.01 and 0.24 mg O_2_ L^−1^, respectively, only a small fraction of the c. 10 mg O_2_ L^−1^ needed to oxidize the H_2_S present. Another potential oxygen debt is build-up of dissolved organic matter (DOM) in waters below the halocline. Such build-up could be caused both by long-term accumulation of legacy DOM from increased sedimentation and by oxygen deficiency slowing the decomposition of organic matter. Inflow of warmer surface water could potentially add DOM, as well as increase its rate of decomposition. Much DOM is however likely to be of terrestrial origin and largely refractory. Data for evaluating this are scarce and DOM decomposition rates in Baltic Proper deep waters are not well studied.

The quick return of hypoxia in the deep water after recent inflows is likely to be caused by NH_4_ and H_2_S remaining after long stagnation periods combined with the successively decreased oxygen store below the halocline over the last 30 years (Fig. 5). The phosphate released from sediments during hypoxia is often assumed to be a legacy of long-term eutrophication (e.g. Andersen et al. 2017). It is however difficult to differentiate between the relative importance of potentially increased oxygen consumption from decomposition of legacy organic matter in the sediment, the eutrophication legacy in the form of large $\Sigma$OD and a highly depleted oxygen store below the halocline and a changed inflow regime, with longer stagnation periods.

### Distribution of oxygen debt between depth strata and basins

**Fig. 7:**
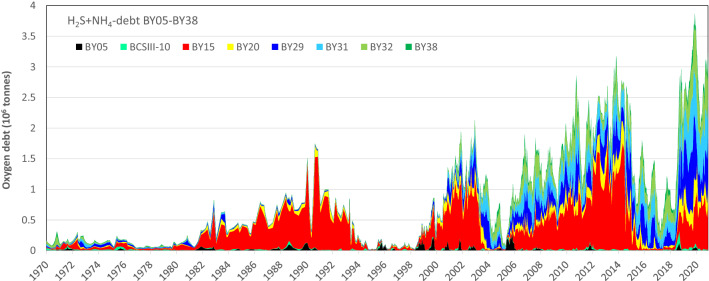
Time series of summed daily $\Sigma$OD (H_2_S + NH_4_ oxygen debt) per basin. After 2000, most of the oxygen debt has gradually shifted to the northern basins

**Fig. 8:**
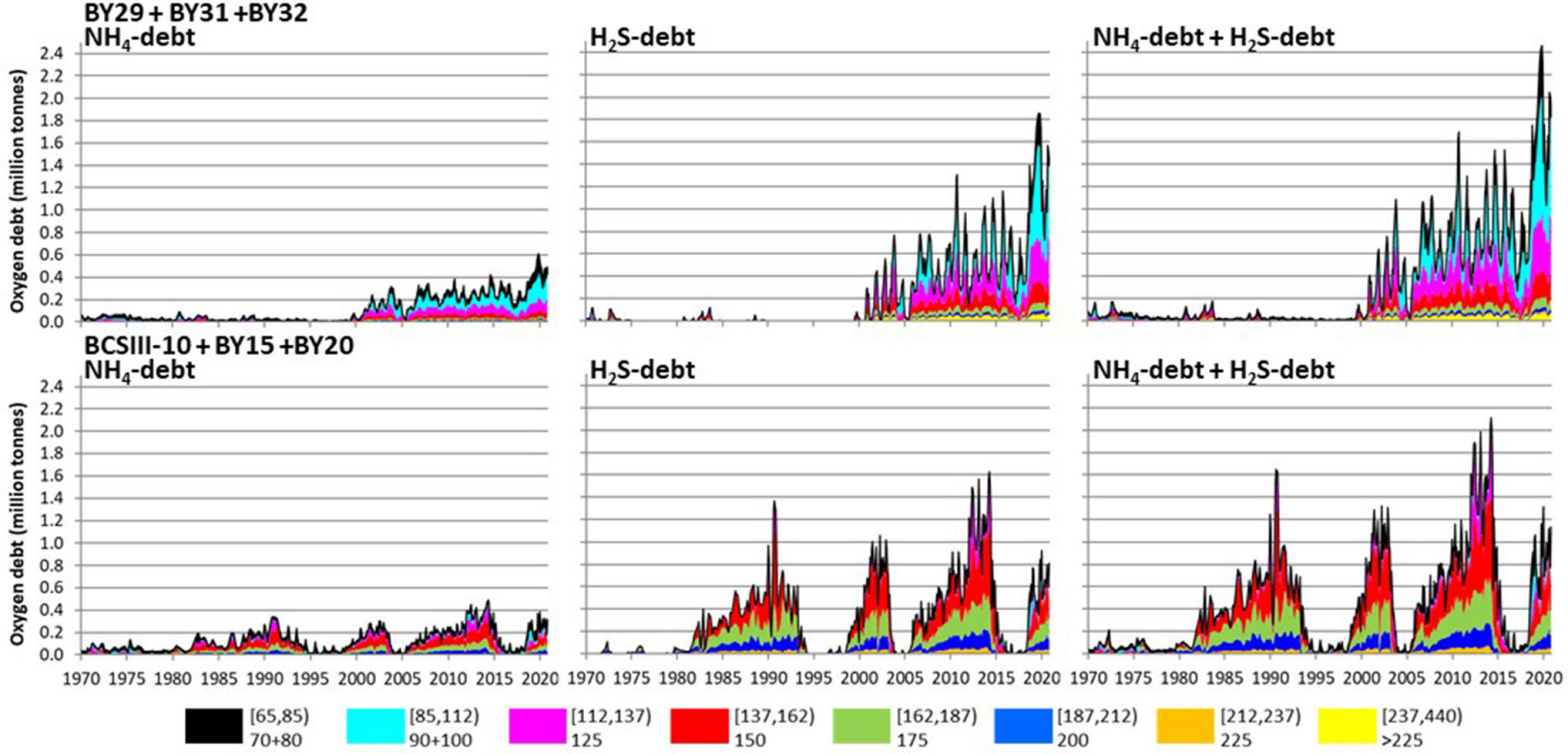
Time development of the depth distribution of NH_4_-, H_2_S- and total oxygen debts (left, centre and right panels) in tonnes oxygen in the northern basins (BY29 + BY31 + BY32, upper panels) and the central basins (BSCIII-10 + BY15 + BY20, lower panels). Colours indicate depth strata and depth of measurement. Depth strata have been combined to approximately equal depth intervals (20–25 m)

Historically, oxygen debt in the Baltic Proper has been dominated by conditions in the central basin BY15 (Fig. 7). Prior to the millenium shift, this basin contributed 60 to 90% of the total oxygen debt below 65 m during periods of severe oxygen debt. Most of this oxygen debt was found in the 150 and 175-m depth strata (137.5–187.5 m). The total oxygen debt ($\Sigma$OD) in the central basin BY15 peaked at the end of the 2006–2014 stagnation period, and was not exceptionally high in 2020. In the northern and northwestern basins (BY29, BY31 and BY32), $\Sigma$OD increased rapidly after 2000, possibly due to displaced water from the central basins (BCSIII-10, BY15, BY20). The overall oxygen debt also became more evenly distributed in the watermass. In periods of high oxygen debt the quantitatively greatest debt is now found nearer the halocline at 75–137.5 m (Fig. S6). In the most recent stagnation period (since 2017), the distribution of oxygen debt has shifted even more from the central basins to the northern and northwestern basins. The latter basins were previously comparatively well oxygenated but oxygen debt is now found relatively close to the halocline, with ~ 70% of the total oxygen debt now found in the 90 to 125-m depth strata (Fig. 8).

It is worrying that oxygen debt is now found shallower, affecting increasing bottom areas. When sediments turn anoxic, large-scale internal loading of phosphorus occurs as iron is reduced and bound to sulphide, loosing its ability to bind phosphate. Expanding hypoxic sediment areas increase phosphate release to the overlying water, potentially aggravating eutrophication. Oxygen debt closer to the halocline also increases the likelihood of water with low oxygen content penetrating into coastal areas during upwelling events. Water with low oxygen and high phosphate concentration now reaches the northernmost basins of the Baltic Proper and increasingly also the Gulf of Bothnia (Rolff and Elfwing 2015). On the other hand, the northern basins of the Baltic Proper are its most wind-exposed areas and the continuous exchange through minor inflows causes more water exchange in the depth strata close to the halocline than at greater depth (Stigebrandt 1987; Holtermann et al. 2020). Vertical mixing and import of oxygenated water in the northern basins could thus potentially be more effective in reducing oxygen debts at shallower depths than in the deep areas of the central basin.

### Seasonal maximum of oxygen debt

**Fig. 9:**
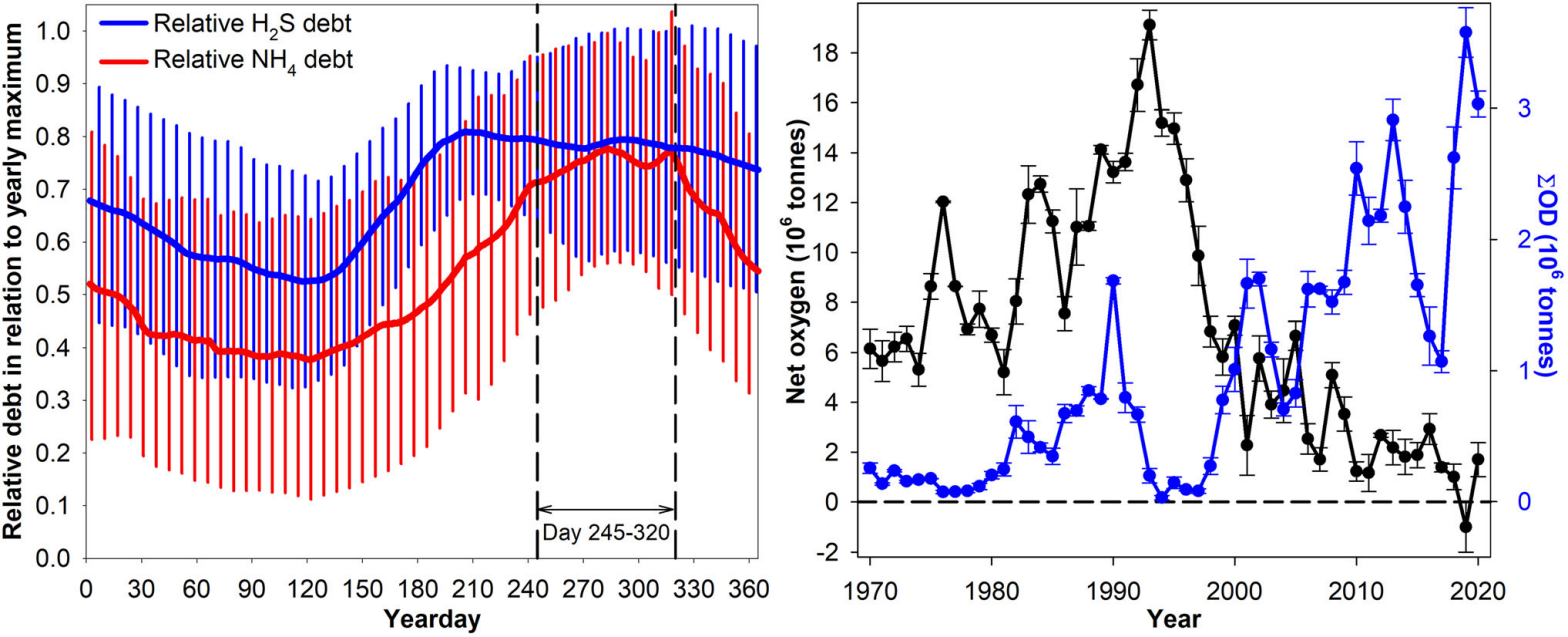
Daily averages and weekly standard deviation of H_2_S and NH_4_ oxygen debts as proportion of annual maximum debt for the period 1994 to 2019 in basins BY05 to BY38 (left panel). Yearly net oxygen (oxygen present minus H_2_S and NH_4_ oxygen debts) and $\Sigma{\text {OD}}$ in the same basins as average and standard deviation for day 245–320 in 10^6^ tonnes (right panel)

The lowest oxygen concentration of the year is often more critical to animals than the mean. This is particularly true for benthic species with limited ability to migrate to areas with sufficient oxygen. Some benthic organisms can survive short periods of severe oxygen deficiency by entering a state of low metabolic activity, but there are limits. From a biological point of view it is therefore important to consider the annual maximum of the yearly oxygen debt as a time trend. To identify when in the year H_2_S and NH_4_ debts peaked in waters below 65 m, all daily values for each of the years 1994–2019 (with sampling at monthly or shorter intervals) were normalised to the maximum debt of the corresponding year. The daily averages of these normalised values indicated the year-days 245 to 320 (1 Sept–15 Nov) to be a good average estimate of the time of the seasonal maximum oxygen debt for both H_2_S and NH_4_ (Fig. 9).

In the latest period of oxygen depletion (2016–2020), the maximum annual average of H_2_S oxygen debt was ~ 2.5 million tonnes and the corresponding NH_4_ oxygen debt ~ 1 million tonnes during days 245 to 320. Peak values for the preceding period (2004 to 2014) were around 2 and 0.7 million tonnes for H_2_S and NH_4_ oxygen debt, respectively, during day 245 to 320 (Fig S7). The annual maximum debt means were up to 15% higher than the straight yearly average. The difference between the average maximum $\Sigma{\text {OD}}$ (day 245–320) and the minimum (day 60–120) was on the order of half a million tons for basins BY05-BY38. The seasonal debt increase occurred almost entirely between 65 and 112 m depth (70 to 100 m estimates), most likely due to decomposition of settling organic matter. As $\Sigma{\text {OD}}$ peaked, the minimum net oxygen (oxygen present reduced by H_2_S and NH_4_ oxygen debts) became negative in 2019 (Fig. 9).

### Oxygen loss per area and volume

The oxygen decrease during stagnation can be calculated by regression from net oxygen showed by the black line in Fig. 5. The deep water (B65) oxygen decrease, calculated for six periods of rapidly decreasing oxygen (Table 2), varied between 1.4 and 5.2 million tonnes per year. The regression slopes estimate the net loss of oxygen and not consumption. Supply and consumption cannot be separated with the method used here, since oxygen will also have been transported to waters below the halocline by summer inflows, minor winter inflows as well as by an unknown amount of storm-driven vertical mixing. The bottom area underlying depths > 65 m in basins BY05-BY38 is ~ 86 700 km^2^ and the volume ~ 3 500 km^3^. The annual net losses of oxygen in basins BY05-BY38 are thus 16–59 g m^−2^ year^−1^ or 0.40–1.5 g m^−3^ year^−1^.

**Table 1:** Basin volumes and their relative contribution to the total volume of water below 65 m

Basin	Volume (km^3^)	Part of total vol. (%)
BY5	147	4.2
BCSIII-10	351	10.1
BY10+BY15	1174	33.7
BY20	351	10.1
BY31	389	11.2
BY29	607	17.4
BY32	299	8.6
BY38	72	2.1
LL12	52	1.5
LL7	37	1.1
LL3	6	0.2

**Table 2:** Oxygen loss in B65 of basins BY05 to BY38 during periods of rapid deoxygenation. Tonnes denoted “t”. The first two regressions are strongly affected by a changing halocline depth

Start date to End date	Regression equation and R^2^	Oxygen lost		
	(slope in tonnes O_2_ day^−1^)	(10^6^ t year^−1^)	(g m^−3^ year^−1^)	(g m^−2^ year^−1^)
1984-01-15 to 1986-09-08	*y* = 2.58E8−7 887.69*day (0.89)	2.9	0.83	33
1993-03-25 to 1999-11-15	*y* = 2.48E8−6 644.28*day (0.87)	2.4	0.70	28
2000-03-25 to 2001-11-20	*y* = 5.27E8−14 106.48*day (0.77)	5.2	1.5	59
2005-04-09 to 2007-11-04	*y* = 2.65E8−6 677.06*day (0.69)	2.4	0.70	28
2008-04-09 to 2010-10-11	*y* = 2.28E8−5 591.82*day (0.74)	2.0	0.59	24
2016-01-01 to 2019–10-29	*y* = 1.69E8−3 850.34*day (0.49)	1.4	0.40	16

Meier et al. (2018) tabled previous estimates of Baltic oxygen consumption rates for different periods, basins and depths and found values from 5 to 75 g m^−2^ year^−1^. Their estimate for depths below 230 m in the Eastern Gotland basin was 11 g m^−2^ year^−1^ for stagnation periods during the years 1964 to 2015. For the deep water of the Bornholm basin oxygen consumption has been estimated to 75 g m^−2^ year^−1^ (Stigebrandt and Kalén 2013). The estimates found in this study for net oxygen (oxygen reduced by both H_2_S and NH_4_ debt) loss in B65 in the combined basins BY05-BY38 are comparable to some of the consumption rates compiled by Meier et al. (2018) for water below the halocline but considerably higher than 11 g m^−2^ yr^−1^. The net losses of oxygen found in our study were for periods of rapid deoxygenation following oxygenation by inflows (Table 2) and are therefore likely to be high estimates. A field study after the 2014 MBI found oxygen fluxes between sediment and water of 7 and 15 mmol m^−2^ day^−1^ of oxygen at 171 and 210 m respectively in the central basin BY15 (Hall et al. 2017), corresponding to 82 to 175 g m^−2^ year^−1^. Presumably the amounts of organic material at these stations were higher than in shallower areas, due to sediment focussing and previous oxygen deficiency.

Carstensen et al. (2014) introduced a variable called total apparent oxygen utilization (TAOU) and TAOU flux, which to our understanding estimates the total loss of oxygen below the lower limit of the halocline. Their results are, however, difficult to compare to ours, since their calculations use a variable halocline depth and make assumptions on the vertical transport and effects of external nutrient load on oxygen consumption.

### How much oxygen is required to reach predefined target levels for oxygen conditions?

To set reasonable limits for evaluating the oxygen status of the Baltic Sea it is important to consider how much oxygen would be required to reach a specific target. Elimination of $\Sigma{\text {OD}}$ (all H_2_S and NH_4_ present) could be set as an environmental target for a desired oxygenation state. Alternatively, a more ambitious target for oxygenation state could be set, e.g. “at least 2 mL L^−1^ oxygen all the way to the bottom” or some other target that requires additional oxygen beyond elimination of H_2_S and NH_4_, but this will drastically increase the demand for new oxygen.

**Fig. 10:**
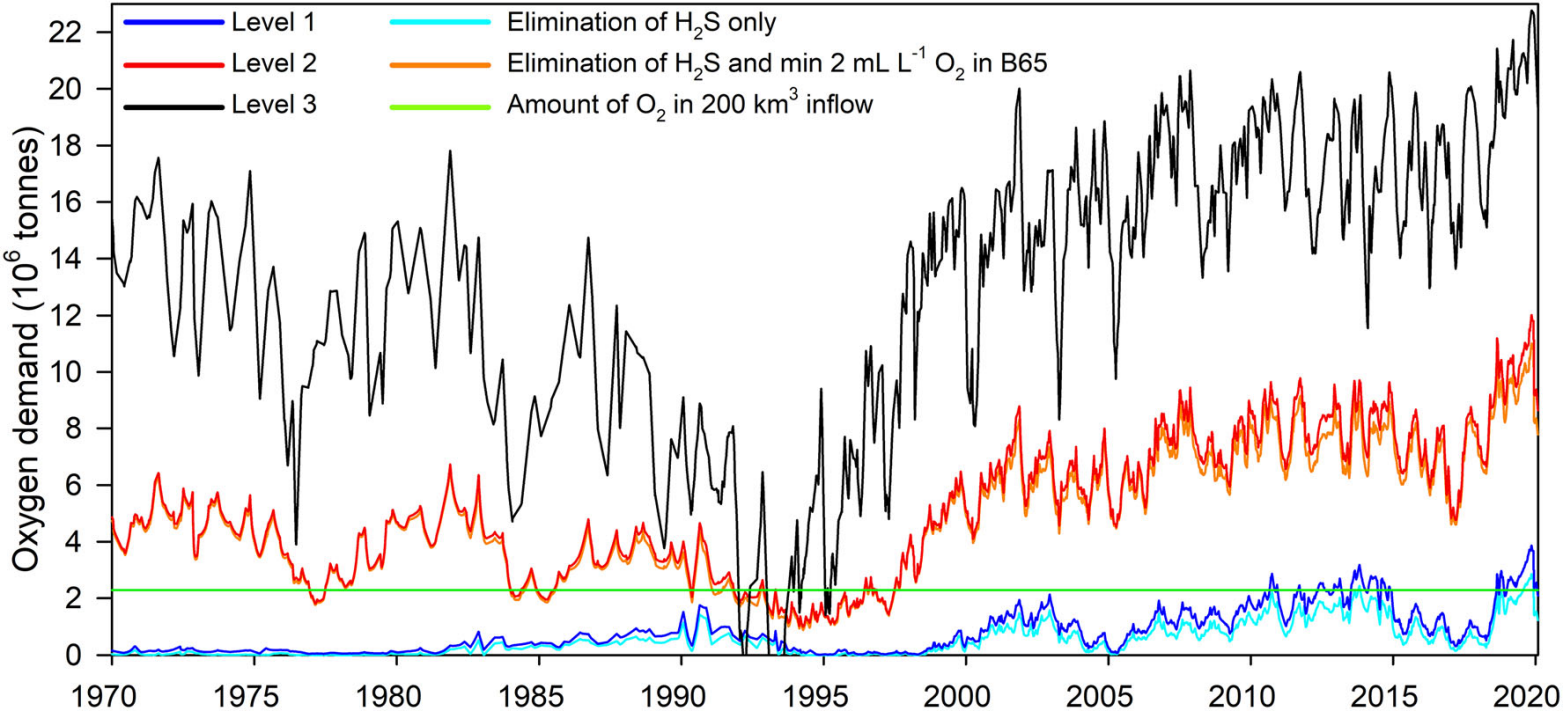
Time development of oxygen demand for reaching 3 levels of hypothetical targets for oxygen status. The approximate amount of oxygen in a large inflow of 200 km^3^ with 8 mL L^−1^ oxygen is indicated by the green line. Level 1: Elimination of all H_2_S and NH_4_ present (the same elimination target for H_2_S only is also indicated). Level 2: Elimination of all H_2_S and NH_4_ present and attaining a minimum of 2 mL L^−1^ of oxygen in all water below 65 m (the same elimination target for H_2_S only is also indicated). Level 3: Attaining the average oxygen conditions in 1993

We estimated how much oxygen would be required to reach three hypothetical target levels for B65. Level 1: Elimination of all H_2_S and NH_4_ ($\Sigma{\text {OD}}$) present, Level 2: Elimination of $\Sigma{\text {OD}}$, plus attaining a minimum of 2 mL L^−1^ of oxygen in all water below 65 m, Level 3: Attaining the average oxygen conditions following the 1993 inflow, when oxygen concentration in the central basin was (briefly) close to 3 mL L^−1^ all the way to the bottom (Fig. 10). In winter, the surface oxygen concentration in the Kattegat, close to the Sound, is normally ~ 8 mL L^−1^ (data not shown). An inflow of 200 km^3^ with this concentration contains just over 2 million tonnes of oxygen. Entrainment magnifies the volume of the inflow and may increase the transported oxygen amount, but oxygen is also consumed on the way to the central and northern basins. The 2 million tonnes in a large inflow are therefore only an approximation of potential oxygen transport to the deep areas.

In the past, the content of oxygen in a large inflow was theoretically sufficient to eliminate all H_2_S and NH_4_ debt (Level 1), but this is currently not the case (Fig. 10). If entrainment in the southern basins greatly increases the volume of oxygenated water it might still suffice. To reach 2 mL L^−1^ in all water below 65 m (Level 2) at present requires six times the oxygen (~ 12 million tonnes) present in a large inflow. Reaching the average conditions found in 1993 would require ~ 22 million tonnes, almost 11 times the oxygen amount in a large inflow. The early 90’s appears, however, to have been an exceptional situation, with twice as much oxygen in B65 as in preceding decennia. That infrequent inflows would improve oxygen conditions much beyond eliminating all H_2_S and NH_4_ therefore seems unrealistic at present.

When scaling and motivating measures for eutrophication abatement, it is important to set attainable targets and to have reliable and sensitive estimates of how far from that target the current state is. Measures to reduce nutrient load are costly and the choice of target therefore has economic implications. It is tempting to set the target to a biologically desirable state, such as a deep water oxygen concentrations of at least 2 mL L^−1^, which would allow multicellular animal life. This is, however, likely to be unrealistic, or attainable only for brief periods, since periodic oxygen deficiency is natural in the deepest parts of the Baltic Sea. If a less demanding goal is set, it is still important to be able to evaluate the feasability of reaching it. The bottom areas covered by hypoxia or anoxia, or the corresponding water volumes, give good descriptions of the severity of the problem by indicating the size of the area or volume that is uninhabitable for multicellular animals. It is, however, desirable to have a measure of oxygen status that is sensitive to the rapid changes seen in the deep Baltic Proper (Fig. 6). The measure of bottom areas covered by hypoxic or anoxic water is dampened by the progressively increasing volume of water strata as depth decreases. It therefore does not properly reflect increasing amounts of H_2_S in the anoxic waters. We propose yearly maximum total oxygen debt $\Sigma{\text {OD}}$ and minimum net oxygen (oxygen present minus $\Sigma{\text {OD}}$) as good descriptors of Baltic Proper oxygen status that can be easily calculated for individual or combined basins, and are based directly on monitoring stations with long and reliable time series. It is however unclear how this, or any other measure of oxygen status, scales to Baltic Sea nutrient load, since whilst the load has decreased substantially in the past three decades, oxygen status has deteriorated (Krapf et al. 2022).

## Conclusions

The Baltic Proper is presently in a phase of rapid changes in oxygenation status. Inflows that previously could oxygenate the deep water now give only short-lived improvements of oxygen conditions in the water below the halocline. The extent to which this is caused by the accumulated H_2_S and NH_4_ oxygen debt, a legacy accumulation of organic material or a changed inflow regime since the early 1980s is unclear.

The oxygen debt caused by accumulation of H_2_S and NH_4_ ($\Sigma{\text {OD}}$) has increased and now partly persists even after a major inflow. In recent years, the net oxygen (oxygen present − $\Sigma{\text {OD}}$) in B65 of the combined major basins (BY05-BY38) has become negative. Most previous studies of the Baltic oxygen debt focussed on H_2_S, but our results indicate that NH_4_ generally contributes about 30% to $\Sigma{\text {OD}}$ and in periods of large oxygen debts up to 50%. Ammonium also appears to persist longer than H_2_S after oxygenating inflows.

The $\Sigma{\text {OD}}$ is dynamic (Fig. 6) and can vary from less than 0.5 million to over 3.5 million tonnes in just a few years. This is not well described by other measures of oxygen status (e.g. areas or volumes of hypoxic and anoxic waters). The depth strata and basins containing the bulk of the $\Sigma{\text {OD}}$ can also change rapidly. The severe $\Sigma{\text {OD}}$ that previously was restricted mainly to the central basins, has since the millennium shift spread to the northern basins. Following the large 2014 inflow, the bulk of the $\Sigma{\text {OD}}$ shifted from the deeper layers of the central basins to intermediate and near-halocline layers in the northern and western basins (Fig. 8). This might result in more effective elimination of the $\Sigma$OD by wind-induced mixing, but also increases the risk of episodic coastal hypoxia by upwelling and of export of hypoxic, nutrient-rich waters to the Gulf of Bothnia.

Some further factors potentially contributing to the oxygen debt are not evaluated in this study but are briefly discussed. Amongst these are temperature, through effects on oxygen saturation and sediment decomposition rate, and potential accumulation of reduced substances other than NH_4_ and H_2_S. The scarce available data suggest iron and manganese contribute only marginally to the debt, but are insufficient for evaluating the effect of a possible long-term DOM accumulation. The net oxygen and $\Sigma{\text {OD}}$ are however indicators of the sum of all these effects. How these variables relate to nutrient load remains unclear.

In setting targets for the oxygen status of the Baltic Proper it is important to consider the feasibility of reaching the targets and to choose suitable indicators for evaluating present status in relation to the target or desired state. Under present conditions, it seems highly unlikely that oxygen concentrations of > 2 mL L^−1^ in the deepest depressions, allowing multicellular animal life in the bottom sediments, will be attainable for extended periods. If we set targets that are highly improbable to reach, even important improvements can be seen as failures, and erode public support for costly mitigation efforts. Considering the present poor oxygen conditions in the Baltic Proper, elimination of most $\Sigma{\text {OD}}$ for extended periods appears as an interesting initial environmental target. The combination of $\Sigma{\text {OD}}$ and net oxygen (oxygen present reduced by $\Sigma{\text {OD}}$) during the annual maximum of oxygen debt are likely to be good, practical estimators of position along the route to a chosen target and beyond.

## Supplementary Information

Below is the link to the electronic supplementary material.

Supplementary file1 (PDF 2294 KB)

## Footnotes

1 https://sharkweb.smhi.se/hamta-data/

2 https://www.ices.dk/data/Pages/default.aspx

3 http://archive.iwlearn.net/helcom.fi/GIS/BalanceData/en_GB/main/index.html

4 http://data.bshc.pro/
