# Supplementary material for: How oxygen deficiency in the Baltic Sea proper has spread and worsened: The role of ammonium and hydrogen sulphide
Source: Ambio. 2022 Jun 23;51(11):2308–24. doi: 10.1007/s13280-022-01738-8 (PMC9481832; doi:10.1007/s13280-022-01738-8)
Supplement: Supplementary file 1 — Supplementary file1 (PDF 2294 KB) [file 13280_2022_1738_MOESM1_ESM.pdf]

Ambio

Electronic supplementary material
This supplementary information has not been peer reviewed.

Title: **How oxygen deficiency in the Baltic Sea proper has spread and worsened: The role of ammonium and hydrogen sulphide**

Electronic Supplement

Additional information on methods

One of the reasons for using data from only two original sources is that it reduces the risk of false time trends and variation due to changes in sampling and analytical methods within and between different operators. Details of sampling and analytical methods are available from the national data hosts. Older oxygen data were generally measured by Winkler analysis, later data also by optode, checked by Winkler analysis at specific depths and periodically at all depths, with good agreement between methods.

Since station BCSIII-10 was not sampled before 29 Oct 1980, data from a station BY9 (lat. 56.1, long. 19.3), northeast of BCSIII-10, was used before then. Station BY9 is ~120 metres deep, whereas BCSIII-10 is only ~90 metres deep. In the period using data from BY9, data from 90- and 120 metres depth at BY9 was used to correspond to the 80 and 90 metres (maximum) depths at BCSIII-10. This introduces a minor underestimate of ammonium and hydrogen sulphide after 1980 in the deepest waters of basin BCSIII-10.

Each profile for each variable was inspected visually and compared to the profiles of the two previous and two subsequent sampling occasions and to the corresponding profiles from the preceding and following year. Values missing for a depth were generally linearly interpolated from surrounding depths. If values were missing for several adjacent depths, they were interpolated from the profiles of the nearest neighbours in time, if reasonably close (in general a month in either direction). The validity of uncertainties in oxygen profiles were evaluated using other variables (e.g. presence of ammonium, oxidised inorganic nitrogen, change in salinity or temperature by displacement of water masses etc.). Data were only excluded (approximately 1 % of data) if there were strong reasons to do so and were not smoothed. If a profile was too incomplete to be reliably completed by interpolation it was not used. The highly dynamic results are therefore a reflection of the true variation in the raw data.

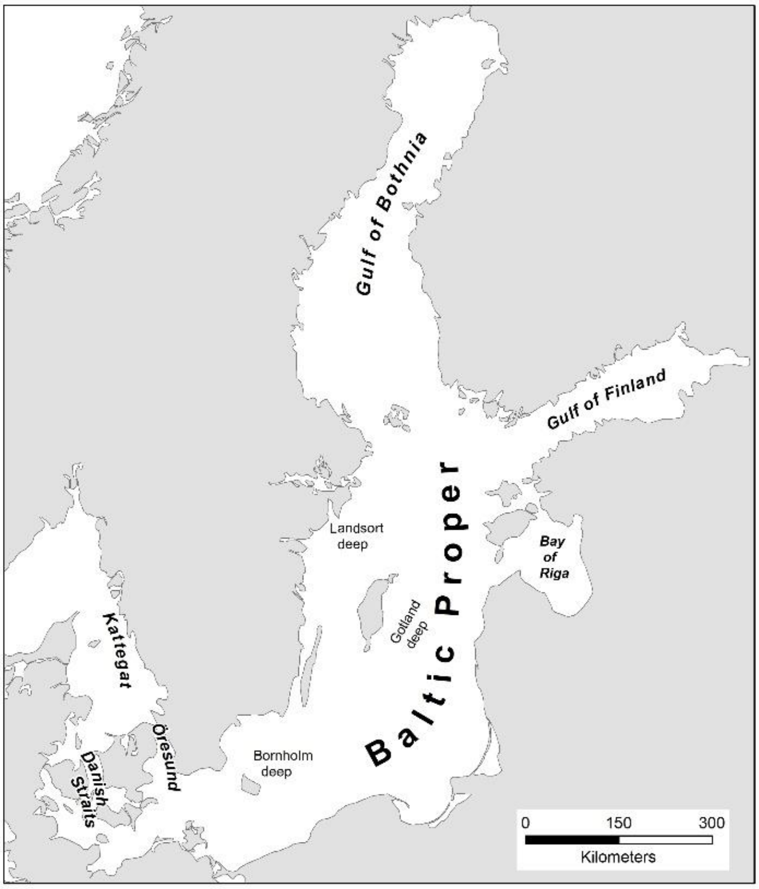

Fig S1 Map of the Baltic Sea.

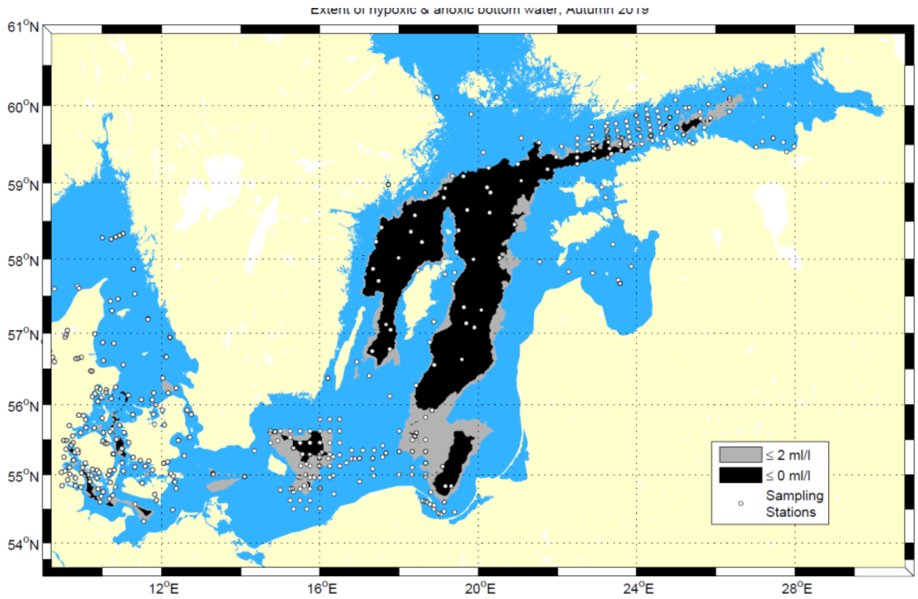

Fig S2 Map of present hypoxic and anoxic bottom areas in the Baltic Proper 2019. Reproduced from Appendix 2 in Hansson, M., L. Viktorsson, and L. Andersson. 2019. Oxygen survey in the Baltic Sea 2019 - Extent of anoxia and hypoxia, 1960-2019. Swedish Meteorological and Hydrological Institute.

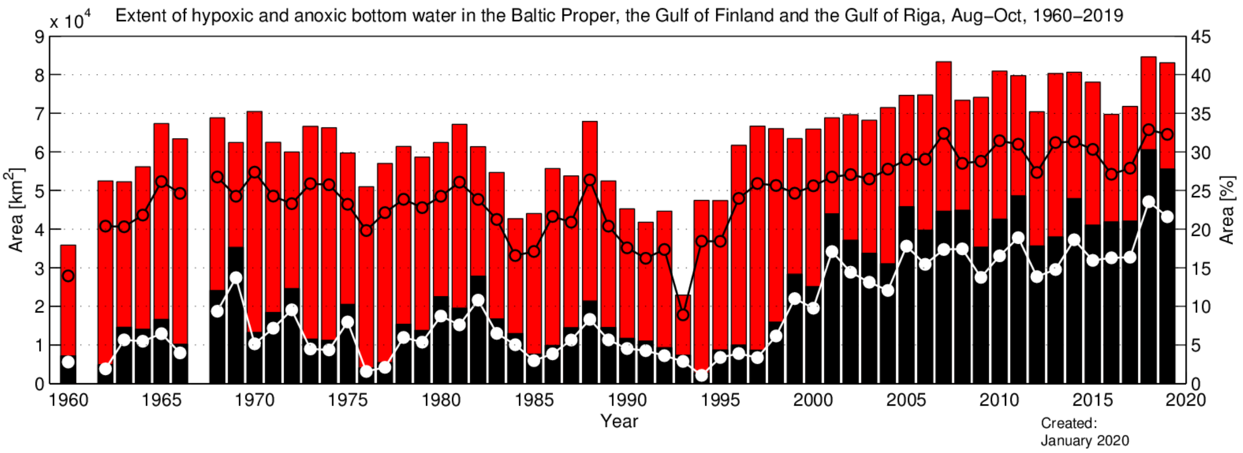

Fig S3 Total bottom area covered by anoxic (black) and hypoxic water (red) on left scale. On right scale percent of area in the Baltic Proper, the Gulf of Finland and the Gulf of Riga covered by anoxic water (white circles) and hypoxic water (red circles). Reproduced from Figure 3 in Hansson, M., L. Viktorsson, and L. Andersson. 2019. Oxygen survey in the Baltic Sea 2019 - Extent of anoxia and hypoxia, 1960-2019. Swedish Meteorological and Hydrological Institute.

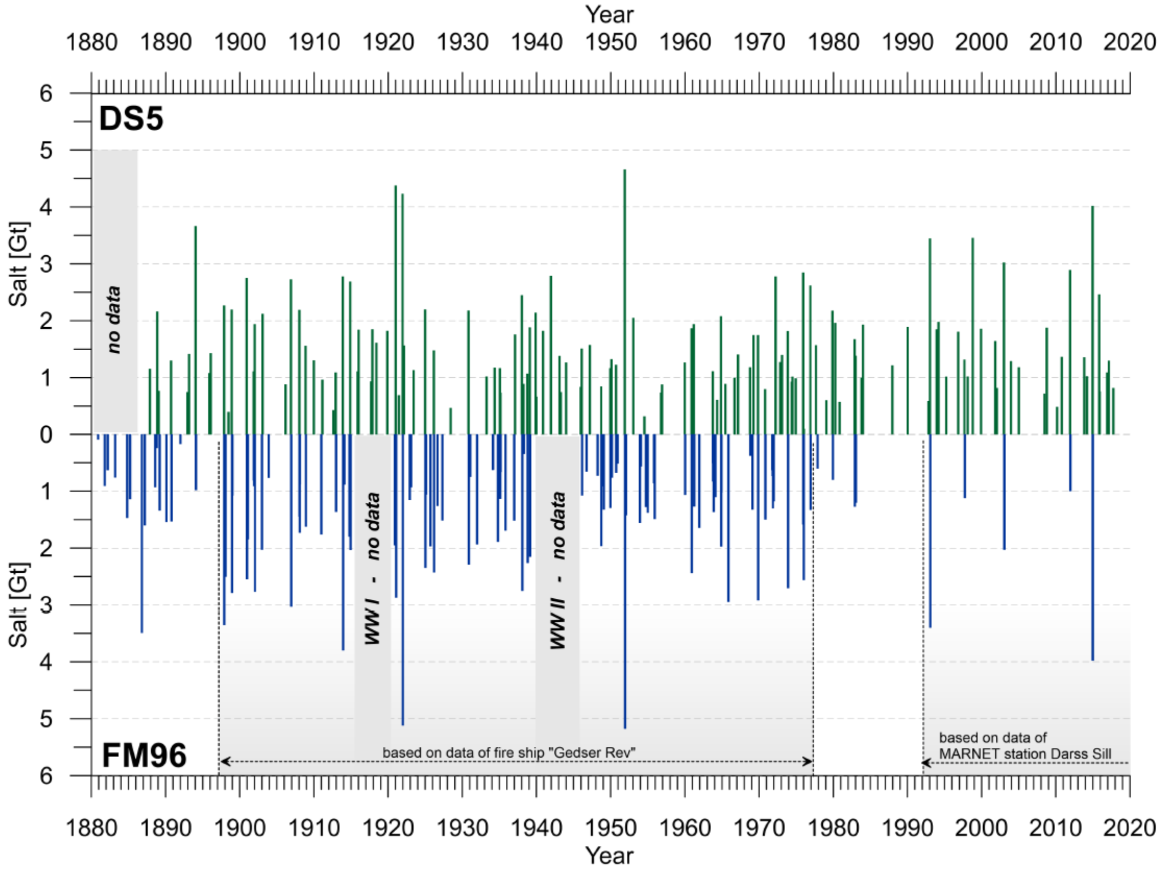

Fig S4 Major Baltic Inflows (MBIs) estimated as Gtonnes salt by two datasets (DS5 & FM96). The green columns (upper panel) is a revision of the blue columns (lower panel). Reproduced from figure 9 in Mohrholz, V. 2018. Major Baltic Inflow Statistics - Revised. Frontiers in Marine Science 5: 384.

Station	Latitude (DD)	Longitude (DD)	Approx. maxdepth	Corresponding basin
BY1	55.0	13.3	46	BY01
BY2	55.0	14.1	48	BY02
BY5	55.2	16.0	91	BY05
BCSIII-10	55.6	18.4	91	BCSIII-10
BY15+BY10	57.3	20.1	242	BY15
BY20	58.0	19.9	199	BY20
BY29	58.9	20.3	180	BY29
BY31	58.6	18.2	457	BY31
BY32	58.0	18.0	203	BY32
BY38	57.1	17.7	111	BY38
LL12	59.5	22.9	83	LL12
LL7	59.8	24.8	84	LL7
LL3A	60.1	26.3	68	LL3

Table S1 Position and maximum depth of stations and their corresponding basins

Pathway					N to O molar ratio	N to O mass ratio
**Aerobic**						
$NH_4^+ + 1.5\ O_2$	→	$2H^+ + NO_2^- + H_2O$				
		$NO_2^- + 0.5\ O_2$	→	NO_3^-	1 N : 2 O_2	14:64
						(1:4.57)
**Aerobic+Anammox**						
$NH_4^+ + 1.5\ O_2$	→	$2H^+ + NO_2^- + H_2O$				
Anammox		$NO_2^- + NH_4^+$	→	$N_2 + 2\ H_2O$	2 N : 1.5 O_2	28:48
						(1:1.71)

Table S2 Calculation of oxygen demand for oxidizing NH4 (mass proportions in brackets)

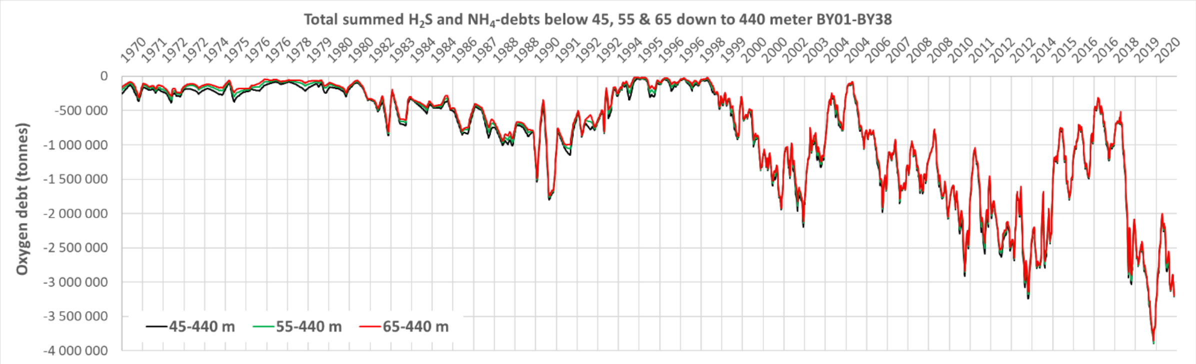

Fig S5 Effects on total H_2S and NH_4 debts (ΣOD, shown as negative oxygen) of using 50, 60 and 70 metre concentrations (45, 55 and 65 metres to bottom) as the upper limits for calculating debts. Differences were negligible.

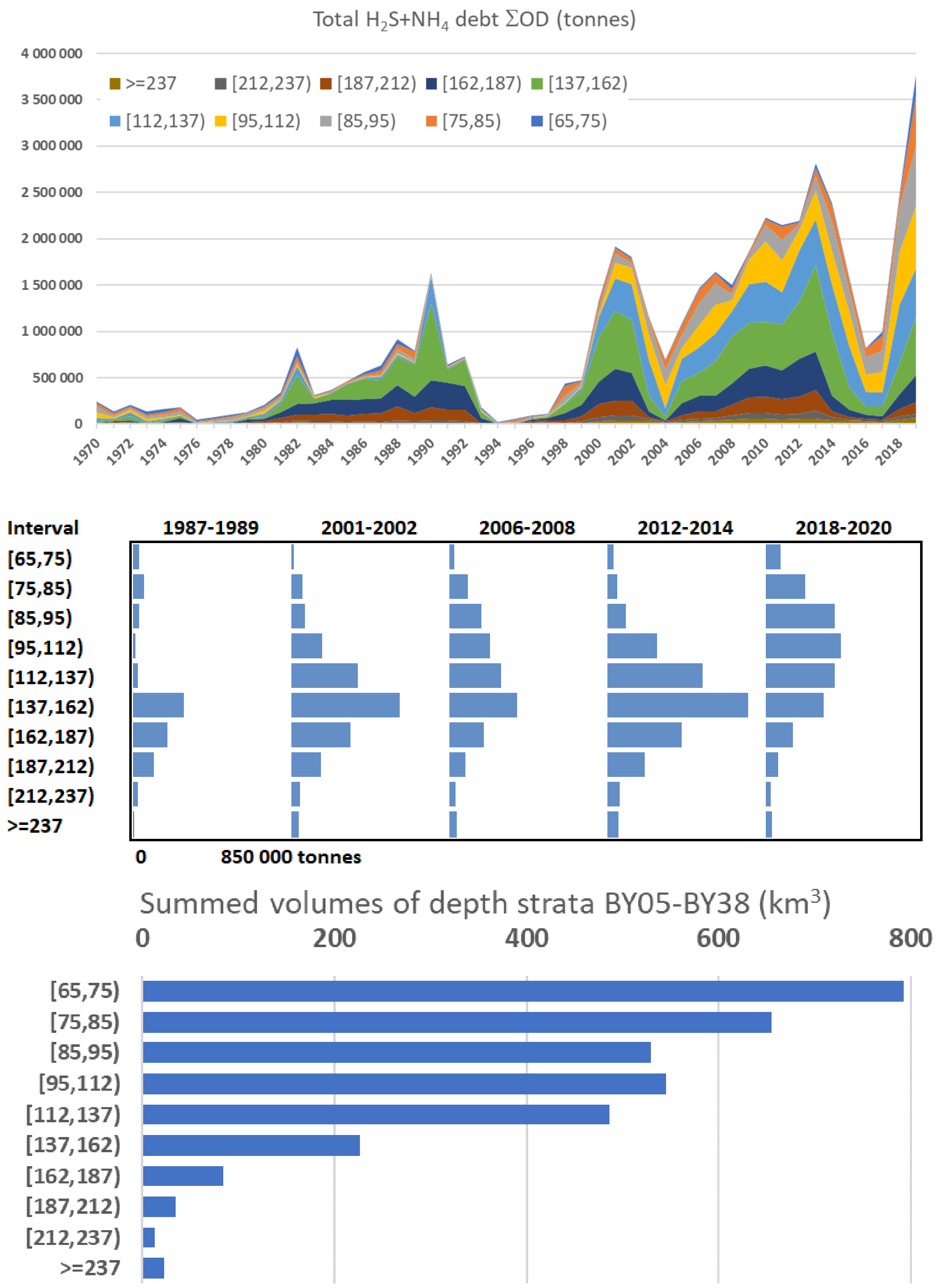

Fig S6 Upper panel: Annual averages of total oxygen debt (ΣOD) in tonnes during oxygen minimum (day 245-320, 1 Sept to 15 Nov) by depths strata below 65 metres (B65) in basins BY05 to BY38. Note that the depth strata are 10 metres down to 100 metres and 25 metres below that.

Middle panel: Depth distribution of summed average ΣOD in tonnes during minimum for five periods of high oxygen debts (same scale for each period (0-850 000 tonnes), in basins BY05 to BY38.

Lower panel: Water volume of depth strata below 65 m for BY05-BY38.

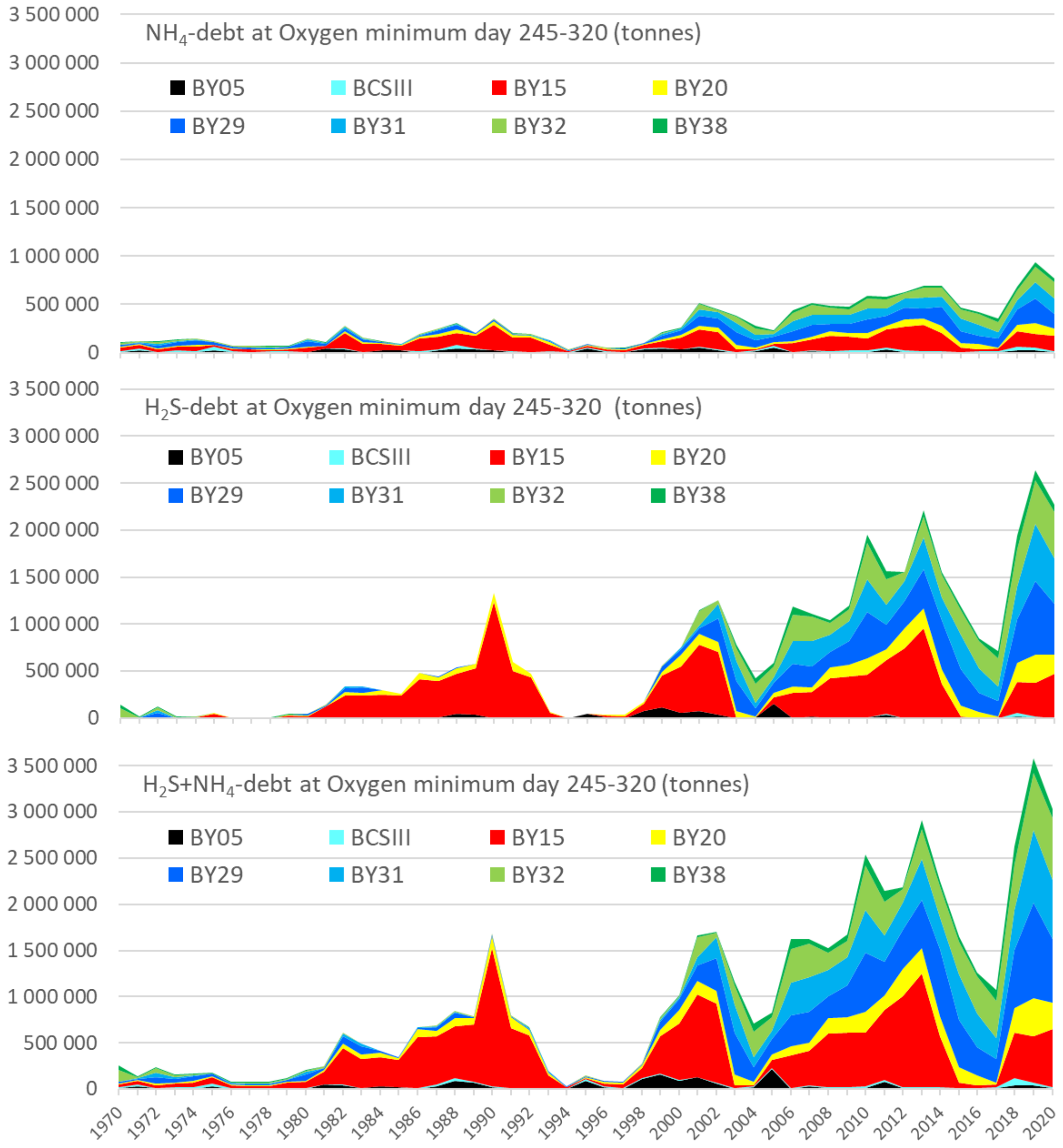

Fig S7 Average annual oxygen debts by basins during oxygen minimum (year-days 245 to 320).
